# Attitudes and Beliefs towards Rotavirus Vaccination in a Sample of Italian Women: A Cross-Sectional Study

**DOI:** 10.3390/vaccines11061041

**Published:** 2023-05-30

**Authors:** Giuseppe Di Martino, Riccardo Mazzocca, Laura Camplone, Fabrizio Cedrone, Pamela Di Giovanni, Tommaso Staniscia

**Affiliations:** 1 Department of Medicine and Ageing Sciences, “G. d’Annunzio” University of Chieti-Pescara, 66100 Chieti, Italy; tommaso.staniscia@unich.it; 2 Unit of Hygiene, Epidemiology and Public Health, Local Health Authority of Pescara, 65100 Pescara, Italy; 3 School of Hygiene and Preventive Medicine, University of L’Aquila, 67100 L’Aquila, Italy; riccardo.mazzocca@graduate.univaq.it (R.M.); laura.camplone@graduate.univaq.it (L.C.); 4 Hospital Management, Local Health Authority of Pescara, 65100 Pescara, Italy; cedronefab@gmail.com; 5 Department of Pharmacy, “G. d’Annunzio” University of Chieti-Pescara, 66100 Chieti, Italy; pamela.digiovanni@unich.it

**Keywords:** rotavirus, vaccine, attitudes, public health, Italy

## Abstract

(1) Background: Rotavirus is the leading cause of severe diarrhea and dehydration in infants and young children worldwide. Despite the proven benefits of vaccination, vaccine hesitancy and refusal remains a significant barrier to achieving high vaccination coverage in many countries, such as Italy. (2) Methods: An online survey was conducted among women aged between 18 and 50 years from Abruzzo Region, Italy. The survey was composed of two main sections: demographic characteristics and attitudes and knowledge about rotavirus vaccination, based on a five-point Likert scale. Logistic regression analysis was performed to evaluate factors associated with willingness to get the rotavirus vaccination. (3) Results: A total of 414 women were enrolled in the study. Women who were unaware of rotavirus more frequently had a lower education level (university degree 62.5% vs. 78.7%, *p* = 0.004) and reported having no children (*p* < 0.001). About half of the enrolled women thought that rotavirus infection is dangerous (190, 55.6%) and that rotavirus can cause a serious illness (201, 58.8%). Regarding associated factors, women informed by a physician were more likely get a vaccination compared to women informed by friends or relatives (OR 34.35, 95% CI 7.12–98.98, *p* < 0.001). (4) Conclusions: The present study showed low levels of knowledge and attitudes towards rotavirus vaccination. These results highlight the need for developing and improving additional public education programs for parents.

## 1. Introduction

Rotavirus is a highly contagious pathogen that causes gastroenteritis, a leading cause of severe diarrhea and dehydration in infants and young children worldwide [1]. Rotaviruses are non-enveloped double-stranded RNA (dsRNA) viruses that have a complex architecture of three concentric capsids that surround a genome of 11 segments of dsRNA. Ten different rotavirus species (A–J) have been classified on the basis of sequence and antigenic differences. Species A rotaviruses are the most common cause of infections in children, and they are further classified into different genotypes. Six strains of species A rotavirus generally account for >90% of globally circulating species A rotavirus: G1P, G2P, G3P, G4P, G9P, and G12P [2]. The majority of children will experience rotavirus infection by the age of five years [1]. Although infection occurring during the first months of life causes the most severe disease, the spectrum of illness can range from mild gastroenteritis to dehydration, electrolyte imbalance, and shock [3], The burden of rotavirus-related morbidity and mortality is particularly pronounced in low-income countries where access to clean water, sanitation, and healthcare services may be limited. In these settings, rotavirus infections can result in devastating consequences, leading to prolonged hospitalizations, increased healthcare costs, and additional strain on already burdened healthcare systems. Even in high-income countries, rotavirus continues to pose a significant health and economic burden. In industrialized countries, deaths from rotavirus are rare, although hospital admissions are frequent. Hospital admissions, emergency room visits, and pediatrician consultations associated with rotavirus gastroenteritis contribute to the overall healthcare burden and have implications for healthcare expenditures and productivity losses. Furthermore, the emotional distress experienced by parents and caregivers witnessing their children suffer from rotavirus infection should not be underestimated.

Rotavirus epidemiology exhibits distinct patterns across different regions. In most low-income countries, rotavirus epidemiology is characterized by one or more periods of relatively intense rotavirus circulation against a background of year-round transmission. This continuous exposure to the virus results in a high proportion of children being infected by the age of five. In the other hand, in high-income countries, a winter seasonality has been typically reported [4]. An international multicenter cohort study of rotavirus infection in the first two years of life demonstrated heterogeneity in rotavirus gastroenteritis incidence across sites and found that three different prior infections are able to give 74% protection against subsequent infections [5]. Understanding these variations in rotavirus epidemiology is essential for tailoring vaccination strategies and optimizing disease prevention efforts.

The introduction of rotavirus vaccines has revolutionized the control of rotavirus infections. Currently available rotavirus vaccines are live attenuated and orally administered, containing human or animal strains that replicate in the human intestine to elicit an immune response. Clinical trials have shown that these vaccines significantly reduce the incidence of severe rotavirus gastroenteritis, hospitalizations, and associated complications. In fact, two oral live attenuated rotavirus vaccines have been authorized and commercialized in Italy for the prevention of rotavirus infection: RotaTeq^®^ (MSD Canada Rahway, NJ USA Inc.) and Rotarix^®^ (GlaxoSmithKline Brentford, UK). RotaTeq^®^ is a pentavalent human-bovine reassortant vaccine and it requires three doses to confer immunization. Rotarix^®^ is a monovalent human rotavirus vaccine and it requires two doses. Both vaccines’ administration should be started before 15 weeks. Rotavirus vaccines have been developed and widely used to prevent this disease and have been shown to be highly effective in reducing the burden of rotavirus-related hospitalizations and deaths [6]. Results from randomized clinical trials were also confirmed in real world data, reporting a substantial protection against rotavirus disease, especially against severe gastroenteritis [7]. The global impact of rotavirus vaccination is clear, showing a 40% reduction in rotavirus prevalence following the introduction of vaccines, as showed by an analysis from 69 countries participating in the Global Rotavirus Surveillance Network [8].

However, despite the proven benefits of vaccination, vaccine hesitancy and refusal remains a significant barrier to achieving high vaccination coverage in many countries [9]. Vaccine hesitancy, driven by a range of factors including safety concerns, lack of awareness, and misinformation, poses a significant barrier to achieving optimal vaccine uptake [10]. Parents’ attitudes and beliefs play a crucial role in vaccine decision-making for their children. Understanding the specific concerns and knowledge gaps among parents regarding rotavirus vaccination is essential for developing targeted interventions to address vaccine hesitancy and improve acceptance rates.

In Italy, the vaccination against rotavirus was included in the vaccination schedule of the National Vaccine Prevention Plan 2017–2019; it is offered free of charge to all newborns in their first year of life. Italy is among the countries where rotavirus vaccination coverage is suboptimal, with reported coverage rates below the recommended threshold of 95% [11,12], indicating the need for further efforts to enhance vaccine uptake. To address this challenge, it is vital to gain insights into the attitudes, beliefs, and knowledge of Italian women, who often play a pivotal role in healthcare decision-making for their children. As part of efforts to improve vaccination uptake and reduce the burden of rotavirus disease, it is essential to understand the attitudes and beliefs of Italian parents towards rotavirus vaccination. This information will help inform public health policies, educational campaigns, and healthcare interventions aimed at increasing rotavirus vaccine coverage in Italy.

In this study, we aimed to evaluate the attitudes and beliefs towards rotavirus vaccination among Italian women, who are often the primary caregivers and decision-makers for children’s healthcare. We conducted a cross-sectional survey of a sample of Italian women of childbearing age, using a structured questionnaire to assess their knowledge, attitudes, and behaviors related to rotavirus vaccination. The study was conducted in Abruzzo region (Southern Italy), which reports a suboptimal coverage for rotavirus vaccination. The findings of this study will provide valuable insights into the current landscape of rotavirus vaccination in Italy and serve as a foundation for designing targeted strategies to improve vaccine coverage and reduce the burden of rotavirus-related diseases in the country.

## 2. Materials and Methods

### 2.1. Study Design and Participants

An online survey was conducted among women aged between 18 and 50 years of age from Abruzzo Region, Italy. The study aimed to investigate the attitudes and beliefs towards rotavirus vaccination among this specific population. Women residing in the Abruzzo Region were eligible to participate in the survey. Convenience sampling was used to enroll participants. The study enrollment period lasted from January 2023 to February 2023.

### 2.2. Survey Administration

Enrolled women were invited to participate in the survey through an online platform. The survey was shared via a QR code invitation on a social network page or directly sent by the investigators through phone contacts. The survey was designed to be anonymous, ensuring participant confidentiality.

### 2.3. Survey Instrument

The survey consisted of two main sections. The first section aimed to gather demographic information and included six items related to participants’ characteristics, such as age, education level, and employment status. Participants who completed this section provided general information about themselves.

The second section focused on assessing knowledge, attitudes, and beliefs regarding rotavirus infection and its vaccination. Participants who reported being unaware of rotavirus infection concluded the questionnaire at this point, while those who were aware of rotavirus proceeded to the next section. This section comprised five items, with the first two items exploring participants’ knowledge of rotavirus infection and the remaining three items examining their beliefs regarding rotavirus vaccination. Each item was measured on a 5-point Likert scale, ranging from 1 (totally disagree) to 5 (totally agree). Responses scoring 4 or 5 were considered as agreement, while scores between 1 and 3 indicated disagreement. Additionally, the final item in this section assessed participants’ willingness to vaccinate their child with a rotavirus vaccine.

### 2.4. Sample Size Estimation

The sample size estimation was based on the expected positive attitude to vaccinating their children with the rotavirus vaccine of 80% [12], with a confidence interval of 95%, an alpha error of 5%, and a power of 80%. Assuming a response rate of 80%, the final expected sample size was calculated to be 384 participants.

### 2.5. Statistical Analysis

To evaluate the reliability of the 5-point Likert scale section, the questionnaire was sent to a training sample of 30 women. This section showed a good internal consistency of 0.801, with a Cronbach’s alpha coefficient of 0.801. Descriptive statistics were used to summarize continuous variables, reported as mean and standard deviation (SD) or median and interquartile range (IQR) based on their distribution. Categorical variables were presented as frequencies and percentages.

Comparisons between groups, such as mothers and childless women or women who accepted or refused rotavirus vaccination, were performed using appropriate statistical tests. Continuous variables were compared using the Mann–Whitney test, while categorical variables were analyzed using the Chi-Square test or Fisher’s exact test. Logistic regression analysis was conducted to identify factors associated with the willingness to receive rotavirus vaccination, with associations expressed as odds ratios (OR) along with 95% confidence intervals (CI). Statistical significance was set at a *p*-value of less than 0.05. The analysis was conducted using STATA v.14 (StataCorp LLC, College Station, TX, USA).

This comprehensive methodology allowed for the collection of data on participants’ demographic characteristics, knowledge about rotavirus infection, attitudes towards vaccination, and the factors influencing their willingness to vaccinate their children against rotavirus. The statistical analysis aimed to identify significant associations and provide insights into the factors influencing vaccination decisions among Italian women in the Abruzzo Region.

## 3. Results

During the study, a total of 414 women were enrolled in the study. The most frequent age category was 30–40 years and the majority of enrolled women were Italian (95.9%), employed (82.6%), with a university degree (75.8%). As reported in Table 1, 342 women (82.6%) were aware of rotavirus infection. Women who were unaware of rotavirus more frequently had a lower education level (university degree 62.5% vs. 78.7%, *p* = 0.004) and were reported to be childless (*p* < 0.001). No differences between mothers and childless women were found regarding age, nationality, and education level. However, mothers were reported to be less frequently employed (75.1% vs. 91.2%, *p* < 0.001).

Among aware women, the majority were informed by a medical physician (63.7%) and most women (63.3%) would get the rotavirus vaccination for their child. The second most represented source of information was friends and relatives (80, 23.4%). The Internet was the third most used source (28, 8.2%).

As regards attitudes, half of the enrolled women thought that rotavirus infection is dangerous (190, 55.6%) and that rotavirus can cause a serious illness (201, 58.8%). The majority of women did not think that rotavirus vaccination is only useful for children attending school or kindergarten (73, 21.3%) and thought that herd immunity can prevent rotavirus infection (71, 20.8%). Despite that, only 202 women (59.1%) thought that the vaccine is effective and safe. In the other hand, 95 women (27.8%) thought that vaccination is more harmful compared to natural infection.

Mother tended to be more fearful about rotavirus infection compared to childless women (scores 4 IQR 3–5 vs. score 4 IQR 3–4, *p* = 0.014). On the other hand, mothers less frequently thought that rotavirus infection can frequently cause hospitalizations (score 3 IQR 3–5 vs. score 4 IQR 3–5, *p* < 0.001). Childless women showed more trust in vaccine safety and efficacy compared to mothers (score 5 IQR 3–5 vs. score 4 IQR 2–5, *p* < 0.001). Significant differences for all items were also reported between women who decided to vaccinate their children for rotavirus and women that refused it, as reported in Table 2. In particular, women that refused rotavirus vaccination reported lower consciousness about infection danger (score 2 IQR 2–3 vs. score 4 IQR 4–5, *p* < 0.001). Additionally, those who refused the vaccination reported lower trust in vaccine safety (score 2 IQR 1–3 vs. score 4 IQR 4–5, *p* < 0.001). Among women with children (221, 53.4%), 106 women reported refusing rotavirus vaccination for their children, mainly for their belief in the useless of the vaccination (47, 44.3%) and their fear of adverse events (44, 41.5%), as reported in Table S1. 

Regarding factors associated with a positive attitude towards rotavirus vaccination, the most significant factor was the channel of information: women informed by physician were more likely to get the vaccination compared to women informed by friends or relatives (OR 34.44, 95% CI 7.23–96.87, *p* < 0.001). Additionally, others channels of information showed greater odds of getting vaccination compared to friends/relatives, as shown in Table 3. Being a mother was also strongly associated with willingness to vaccinate (OR 4.50, 95% CI 2.59–7.8, *p* < 0.001). Unemployed women showed lower willingness to vaccinate their children (OR 0.17, 95% CI 0.04–0.82, *p* = 0.022). Age categories, nationality (OR 0.96, 95% CI 0.16–5.45; *p* = 0.867), and education level (university degree, OR 1.27; 95% CI 0.53–3.67; *p* = 0.654) were not significantly associated with vaccination. Regarding age, despite a non-significant result, we can observe a progressive decrease in the odds ratio, from 0.77 (95% CI 0.30–1.98; *p* = 0.543) to 0.44 (95% CI 0.23–1.76; *p* = 0.223).

## 4. Discussion

The present study was conducted in Abruzzo, an Italian Region with suboptimal vaccination coverages against rotavirus. Results of this study (willingness to vaccinate/vaccination status: 63.3%, Table 1) are in line with data from Abruzzo Region, which reported a low vaccination coverage against rotavirus (58.7% in 2021) [12]. These results showed an improved knowledge and coverage, compared with a similar study performed in Italy during the pre-pandemic period [13,14] that reported a low willingness to vaccinate (15.3%) among women. All enrolled women with previous pregnancy reported having knowledge about rotavirus, so the counseling performed before the first vaccination visit or previous pediatrician visits are likely the main sources of information. In fact, women with previous pregnancy reported the highest level of knowledge, as reported in Table 1: compared to non-mothers, mothers are more conscious about rotavirus danger and about vaccination safety. These results are in line with a previous study, where first time mothers were found to be more hesitant about rotavirus vaccination [15]. Women that refused rotavirus vaccination believed that this vaccine is useless, or that it can be harmful. This can be explained by a low knowledge of rotavirus infection and bad consciousness of adverse events. In particular, the results reported in Table 2 highlighted how mothers have more knowledge about rotavirus but more fear towards vaccination. In facts, mothers disagreed with natural immunity but more frequently thought that vaccination was more harmful compared to vaccination (mothers: 2 IQR 1–4 vs. non-mothers 2 IQR 1–3). As regards vaccine adverse events, several studies demonstrate the safety of the rotavirus vaccine [16,17], in particular in relation to intestinal intussusception, the most feared complication [18]. These results were in line with previous literature: Marchetti et al. performed a web listening analysis of online discussions about rotavirus [19]. They showed that the most relevant factor associated with vaccine hesitancy was the fear of adverse events. However, the main problem highlighted by this study is the false belief that rotavirus infection causes mild illness. Rotavirus gastroenteritis causes a heavy burden on healthcare systems across all Europe, as reported in several studies [20,21]. This high demand for healthcare systems was faced-off with the introduction of the mass vaccination that strongly changed the epidemiology of rotavirus diseases. Several studies reported a decreased incidence in rotavirus infections [22,23,24,25]. These important results were also reported in some regions with low vaccination coverages such as Sicily (Southern Italy): despite low vaccination rates, the incidence of rotavirus related hospitalizations decreased significantly after the introduction of mass vaccination [26]. So, these results highlight the need to improve vaccination coverages. The skepticism about the safety of the rotavirus vaccine and the knowledge about infection severity should be faced off with a strong informative campaign. 

The evaluation of factors associated with vaccine uptake highlighted that socio-demographic characteristics were not significantly associated with the willingness to vaccinate against rotavirus. Despite the lack of statistical significance, a decreasing trend in OR can be observed among age categories. Education level was also not significantly associated with the willingness to vaccinate but, on the other hand, unemployed women were less likely to accept rotavirus vaccination (OR 0.17, 95% CI 0.04–0.82, *p* = 0.022), as reported in Table 3. The multivariate analysis showed that the most important associated factor for vaccine uptake is explication by a physician (OR 34.44, 95% CI 7.23–96.87, *p* < 0.001). Additionally, mothers are more likely to accept vaccination compared to childless women (OR 4.50, 95% CI 2.59–7.8, ***p*** < 0.001). These results are in line with previous literature for other vaccinations [27,28,29]. Maintaining a continuous relationship with the physician or medical institution was positively correlated with receipt of preventive services and it is a point requiring focused activity in order to improve vaccination adherence. Health authorities should improve information campaigns and physicians’ knowledge in order to empower parents’ consciousness about rotavirus vaccination [30]. Specifically, regarding rotavirus vaccination, Marchetti et al. highlighted the importance of the influence of healthcare workers in maintaining parental confidence in vaccination [18], confirming the results of the multivariate analysis. Similar results were reported outside Europe. In particular, pediatricians from the USA are confident in rotavirus vaccination and they recognize its impact on improving health outcomes for children [10]. Unfortunately, also in the USA, many parents perceive that rotavirus vaccination is unnecessary. So, information campaigns should also be implemented among parents or future parents outside healthcare services: the knowledge of parents should be improved to easily achieve better vaccine uptake and to decrease the burden of rotavirus disease.

Additionally, other European countries reported skepticism towards rotavirus vaccination. In France, general practitioners believe that parents see gastroenteritis as a benign disease [31]. Parental mistrust over vaccine safety was also reported in a previous study performed in Greece [32]. However, these studies are not recent, and they were performed when rotavirus vaccination was not refunded in France and Greece, increasing barriers towards vaccine acceptance. 

### Limitations

This is one of the first studies conducted in Italy on the attitude towards rotavirus vaccination. Despite that, these results should be interpreted in light of some limitations: firstly, this study was conducted in a single Italian Region and its results cannot be generalized to the rest of country, where the vaccination coverages can be higher (mean Italian rate in 2021 was 70.4%—[12]). Secondly, the recruitment was carried out via social media, so women that are not confident or non-social network users were excluded. However, we are confident that the majority of Italian childbearing age women usually use social media, so these results should be similar to the entire population of the Region. Finally, the survey reported the vaccination status of children was self-reported by women, so the information is susceptible to recall bias and could lead to an underestimation of the vaccination rate. Additionally, this study was conducted after the pandemic, so the trust towards vaccines in general could be impacted by the COVID-19 period.

## 5. Conclusions

This study reported inadequate knowledge about vaccination and poor levels of learning towards rotavirus vaccination in a sample of Italian women. These results highlight the need to improve education programs for parents. Counseling performed by general practitioners or by public health physicians before the start of the vaccination schedule is a crucial point. Thus, the majority of possible barriers can be overcome with the help of healthcare workers. Informed parents will result in better knowledge, leading to the improvement of vaccination coverage.

## Figures and Tables

**Table 1:** Demographic characteristics of enrolled women.

	Total (n = 414)	Mother (n = 221)	Childless Women (n = 193)	*p*-Value	Unaware about Rotavirus (n = 72)	Aware about Rotavirus (n = 342)	*p*-Value
**Age**				0.985			0.083
<30	101 (24.4)	54 (24.4)	47 (24.4)		11 (15.3)	90 (26.3)	
30–40	282 (68.1)	150 (67.9)	132 (68.4)		57 (79.2)	225 (65.8)	
>40	31 (7.5)	17 (7.7)	14 (7.3)		4 (5.6)	27 (7.9)	
**Nationality**				0.970			0.748 *
Italian	397 (95.9)	212 (95.9)	185 (95.9)		70 (97.2)	327 (95.6)	
Other	17 (4.1)	9 (4.1)	8 (4.1)		2 (2.8)	15 (4.4)	
**Instruction level**				0.887			0.004
University degree or higher	314 (75.8)	167 (75.6)	147 (76.2)		45 (62.5)	269 (78.7)	
High school or lower	100 (24.2)	54 (24.4)	46 (23.8)		27 (37.5)	73 (21.3)	
**Employed**	342 (82.6)	166 (75.1)	176 (91.2)	<0.001	63 (87.5)	315 (92.1)	0.207
**Aware about rotavirus**	342 (82.6)	221 (100.0)	121 (62.7)	<0.001			
**How did you hear about rotavirus?**							
GP/pediatrician						218 (63.7)	
Friends						80 (23.4)	
Internet						28 (8.2)	
others						16 (4.7)	
**Did you get/would you get rotavirus vaccination for you child?**						216 (63.3)	

* Fisher’s exact test.

**Table 2:** Knowledge toward rotavirus and its vaccination.

N = 342	Women in Agreement with the Item (Score 4–5) n(%)	Mothers Median (IQR)	Childless Women Median (IQR)	*p*-Value *	Unwillingness to Get Rotavirus Vaccination Median (IQR)	Willingness to Get Rotavirus Vaccination Median (IQR)	*p*-Value *
Rotavirus infection is dangerous	190 (55.6)	4 (3–4)	4 (3–5)	0.014	2 (2–3)	4 (4–5)	<0.001
Natural immunity is better to gain protection against rotavirus	71 (20.8)	1 (1–2)	2 (1–4)	<0.001	3 (2–4)	1 (1–2)	<0.001
Rotavirus can cause hospitalization and serious illness	201 (58.8)	3 (2–5)	4 (3–5)	<0.001	2 (2–3)	4 (4–5)	<0.001
The vaccination is more harmful compared to natural infection	95 (27.8)	2 (1–4)	2 (1–3)	0.001	4 (3–5)	1 (1–2)	<0.001
The rotavirus vaccine is safe and effective	202 (59.1)	5 (3–5)	4 (2–5)	<0.001	2 (1–3)	5 (4–5)	<0.001

* Mann–Whitney U Test.

**Table 3:** Factors associated with the positive attitude toward rotavirus vaccination.

	OR	95% CI	*p*-Value
**Age**			
<30	ref		
30–40	0.77	0.30–1.98	0.543
>40	0.44	0.23–1.76	0.223
**Italian**	0.96	0.16–5.45	0.867
**University degree**	1.27	0.53–3.67	0.654
**Unemployed**	0.17	0.04–0.82	0.022
**Source of information**			
Friends/relatives	ref		
Physician	34.44	7.23–96.87	<0.001
Internet	4.39	1.33–33.39	0.015
Others	7.22	1.12–63.45	0.009
**Motherhood**	4.50	2.59–7-82	<0.001

OR = odds ratio; CI = confidence interval.

## Data Availability

Data are available after reasonable request to the corresponding author.

## Supplementary Materials

The following supporting information can be downloaded at: https://www.mdpi.com/article/10.3390/vaccines11061041/s1, Table S1: Reasons to refuse the rotavirus vaccination.
