# Supplementary material for: Attitudes and Beliefs towards Rotavirus Vaccination in a Sample of Italian Women: A Cross-Sectional Study
Source: Vaccines (Basel). 2023 May 30;11(6):1041. doi: 10.3390/vaccines11061041 (PMC10302617; doi:10.3390/vaccines11061041)
Supplement: Supplementary file 1 [file vaccines-11-01041-s001.zip › vaccines-2362050-supplementary.pdf]

Table S1. Reasons to refuse the rotavirus vaccination

N=221	
**Did you get rotavirus vaccination for your previous child?**	115(52.0)
**Why not?**	
Useless	47(44.3)
Fear of adverse event	44(41.5)
Over the allowed time	8(7.5)
other	7(6.6)
Did you performed DTP vaccination during pregnancy?	117(52.9)
